# Quantitative analysis of nonsteroidal anti‐inflammatory drugs in dried blood spot from mountain ultra‐trail runners. Contribution of pharmacokinetic models for the interpretation of the results

**DOI:** 10.1002/dta.3781

**Published:** 2024-08-15

**Authors:** Mohammad Shafiq Mashal, Jérôme Guitton, Pierre Sallet, Laurent Bourguignon, Christelle Machon

**Affiliations:** ^1^ Biochemistry and Pharmacology‐Toxicology Laboratory, Lyon Sud Hospital University Hospital of Lyon Pierre‐Bénite France; ^2^ Toxicology Department Pharmacy Faculty of Kabul University Kabul Afghanistan; ^3^ Pharmacology‐Physiology‐Toxicology Department, ISPB Pharmacy Faculty of Lyon University of Lyon Lyon France; ^4^ Association Athlete For Transparency Lyon France; ^5^ Pharmacy Department University Hospital of Lyon, GH Nord Lyon France; ^6^ Analytical chemistry laboratory, ISPB Pharmacy Faculty of Lyon University of Lyon France

**Keywords:** dried blood spot, ibuprofen, mountain ultra‐trail, nonsteroidal anti‐inflammatory drugs, pharmacokinetic model

## Abstract

Monitoring of drug use in athletes is of interest both for health and competition‐related issues. Considering the advantages of Dried Blood Sampling (low invasiveness, easy sampling, long term storage), we have validated a quantitative LC–MS/HRMS method for the screening of 16 nonsteroidal anti‐inflammatory drugs. For all drugs, accuracy and imprecision were within 15% for the 3 levels of quality control and lower than 20% for the lower limit of quantification. Application was performed from samples obtained for Ultra‐Trail du Mont‐Blanc® 2021 and 2022. A focus on ibuprofen and its metabolites (hydroxyibuprofen, carboxyibuprofen, ibuprofen glucuronide and hydroxyibuprofen glucuronide) was made because the results showed that it was the most detected nonsteroidal anti‐inflammatory drug. Further, an interpretation of the ibuprofen concentrations was proposed either from experimental data obtained after an intake of ibuprofen by 10 control subjects, or from a pharmacokinetic modelling and simulations. Depending on the analytical performances of the method, we proposed possible detection windows for ibuprofen in runners. The pharmacokinetic model made it possible to consider two scenarios with and without modification of the total clearance of ibuprofen linked to a modification of the pharmacokinetics of the drugs due to the practice of a long and intense physical activity.

## Graphical abstract

Quantification of 16 nonsteroidal anti‐inflammatory drugs using dried blood spot and LC–MS/HRMS was performed. By using a pharmacokinetic modelling and simulations, and according to the analytical performances of the method, detection windows for ibuprofen in ultra‐trail runners were proposed.

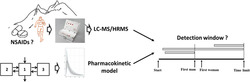

## 1 INTRODUCTION

Several studies have reported the consumption by runners of various drugs during mountain ultra‐trail (MUT).^1^, ^2^ The prevalence varies from one study to another and is ranged from 18% to 70% depending on the method of collecting information: questionnaire or detection from a biological matrix.^1^, ^2^, ^3^ Among compounds, nonsteroidal anti‐inflammatory drugs (NSAIDs) hold a special place because they are the family with the highest prevalence.^2^, ^4^, ^5^ The majority of data about prevalence come from questionnaires but carrying out runner's analysis for the detection of drugs seems relevant because studies have shown an under‐declaration by athletes.^2^, ^4^ Various biological matrices have been used such as oral fluid, blood or urine leading to variable detection windows.^6^ The choice of the biological matrix is also conditioned by the practicability of carrying out the sampling: invasive sampling or not, sample size, need for infrastructure and specialized staff, conditions of the sample transport and stability, … The detection of compounds in the trail runner population brings data on the type of compounds consumed, their prevalence, but also enables to verify the compliance with the rules of the race. The Quartz® program is applied in several MUTs. This program stipulates, among other things, the prohibition of consuming NSAIDs 24 h before and during the race.^7^

The objectives of this work were on the one hand to develop and validate a quantitative analysis method of NSAIDs on a dried blood spot (DBS) sample and on the other hand to interpret the results for ibuprofen in connection with the Quartz® program. For this last purpose, two approaches were performed: one based on experimental data obtained from 10 control subjects, the other one based on pharmacokinetic modelling and simulation. Considering the analytical performances of the developed method, detection windows for ibuprofen in runners were proposed.

## 2 MATERIAL AND METHODS

### 2.1 Chemicals and reagents

The 16 analytical standards of NSAIDs, hydroxyibuprofen, ibuprofen glucuronide and the 6 internal standards (IS) were supplied by LoGiCal® Standards (Luckenwalde, Germany), Sigma‐Aldrich™ (St Quentin Fallavier, France) and Toronto Research Chemicals (Toronto, Canada). Acetonitrile (ACN), methanol (MeOH) both HPLC‐grade were supplied by Biosolve Chemi SARL (Dieuze, France). Acetic acid and chloroform were purchased from Carlo Erba (Val de Reuil, France). Deionized water was provided with an Elga Purelab (Flex system™, High Wycombe, UK). DBS collection device HemaXis DB 10 was supplied by DBS system SA (Gland, Switzerland).

### 2.2 Sample collection

A volume of 10 μL of capillary whole blood was collected at the index finger tip using HemaXis DB 10 device as recommended by the provider. Two spots per trailer were collected. For experiments performed for the method development and the validation, 10 μL of spiked whole blood were deposited onto the DBS paper which was then stored at least 12 h at room temperature before use.

### 2.3 Sample preparation

The day of analysis, the entire spot was cut and transferred into 1 mL of desorption solvent. Four solvents were tested for the desorption of NSAIDs from the paper: MeOH/water (60/40; v/v), ACN/water (60/40; v/v), pure MeOH and pure ACN. After addition of 10 ng of each IS, sample was vortexed and sonicated for 15 min. After removal of the spot, the tube was centrifugated at 13,000 g for 5 min. Then, two protocols were compared. In the first one, the desorption solvent was collected and evaporated under nitrogen. In the second one, a liquid/liquid extraction (LLE) was performed to clean the sample. Thus, after acidification of the supernatant with 50 μL of acetic acid, the LLE was carried by mixing with 500 μL of chloroform. Chloroform was then collected (400 μL) and evaporated under nitrogen. The dry residues were resuspended in 100 μL of the mobile phase (0.1% of acetic acid in ACN/0.1% of acetic acid in water, 25/75, v/v), and 10 μL were injected into the LC–MS/HRMS device.

### 2.4 LC–MS/HRMS analysis

Identification and quantification of NSAIDs and ibuprofen metabolites were performed on an Ultimate 3000 system coupled with a Q‐Exactive Plus Orbitrap mass spectrometer (ThermoFisher Scientific™, Bremen, Germany) as previously described.^6^ Details of the analytical method are presented in Table S1.

### 2.5 Method validation

The validation procedure was conducted in accordance with European Medicine Agency guideline on bioanalytical method validation for the following parameters: carry‐over, selectivity, linearity, accuracy, precision and matrix effect.^8^ For the method validation, all QC samples and calibration standards were prepared by spiking venous whole blood. For the selectivity, the absence of an interfering compound was confirmed by a response of less than 20% of the lower limit of quantification (LLOQ) for each NSAID and 5% for each IS in six blank DBS. The carry‐over was determined by injecting a blank sample after the highest calibration standard. Within‐day accuracy and precision were calculated by analysing six LLOQ and QC samples in a same run. Between‐day accuracy and precision were determined by analysing three LLOQ and QC samples on 3 different days. The LLOQ was considered being the lowest calibration standard with the conditions that accuracy was between 80 and 120% and precision less than 20%. QC samples were considered conformed if the accuracy was between 85 and 115% and the precision less than 15%. The LOD is the concentration at which the signal level of NSAID reaches three times the signal noise. For NSAIDs for which no noise was observed, the LOD was defined as the concentration at which the signal level making it possible to have a chromatographic peak for identification (three scans in full scan mode). The matrix effect was evaluated at two levels (*n* = 6) by comparing the response of a spiked DBS samples after extraction and the response of a sample without matrix.

### 2.6 Pharmacokinetics modelling and simulations

The pharmacokinetic model published by Morse et al. was implemented in Simulx 2021R1 (Lixoft SAS, a Simulations Plus company).^9^ In this model, ibuprofen clearance and volume of distribution were scaled on Normal Fat Mass (NFM), which is calculated with Fat Free Mass (FFM), Total Body Weight (TBW), and a Factor For Fat contribution (FFAT) as follows:

$$\mathrm{NFM}=\mathrm{FFM}+\left(\mathrm{TBW}–\mathrm{FFM}\right)*\text{FFAT}$$

An allometric relationship between NFM and pharmacokinetic parameters was used, with a value of 0.863 for FFAT on clearance, and 0.718 for FFAT on volume of distribution, as reported in the original article.^9^

From this model which includes a standard total clearance (scenario 1), a scenario 2 taking into account a decrease in the ibuprofen total clearance was also generated. In this case, the assumption of a gradual reduction in the clearance as a function of time was modelled by a sigmoid relationship (maximal reduction of 20%, with half of the reduction observed 2 h after the beginning of the trail). A 2bis scenario was also proposed assuming that the trailer took ibuprofen 24 h before the start of the race. During the first 24 h the clearance was not modified, and it only changed when the race started using the same kinetics as scenario 2 (Figure S1).

A virtual population of 1,000 subjects was generated using anthropometrics parameters of a population of long‐distance runners (mean weight: 69.2 kg, mean height: 1.73 m) and a sex‐ratio of 50%.^10^ An oral intake of 400 mg of ibuprofen was simulated, and plasma concentrations were calculated with the 3 scenarios. In all cases, we considered that the trail runners were fasting.

### 2.7 Application

DBS samples were from ultra‐trail runners who participated to the Ultra‐Trail du Mont‐Blanc® (UTMB®) in August 2021 and 2022. UTMB® used the Quartz program event to protect runner's health and to contribute to a cleaner sport.^7^ The collection of the DBS samples was performed just after the finish of the race.

In addition, 10 control subjects (five women and five men) took 400 mg of ibuprofen orally 2 h after breakfast and DBS samples were collected at 24, 36, 48 and 60 h after the intake. During the 60 h following the ibuprofen intake, the 10 subjects did not practice sports.

The agreement of the French ethical committee (Id number: 2021‐A01751‐40, CPP Sud Méditerranée I 13/07/2021) was obtained for the sampling.

## 3 RESULTS

### 3.1 Sample preparation and method validation

**FIGURE 1:**
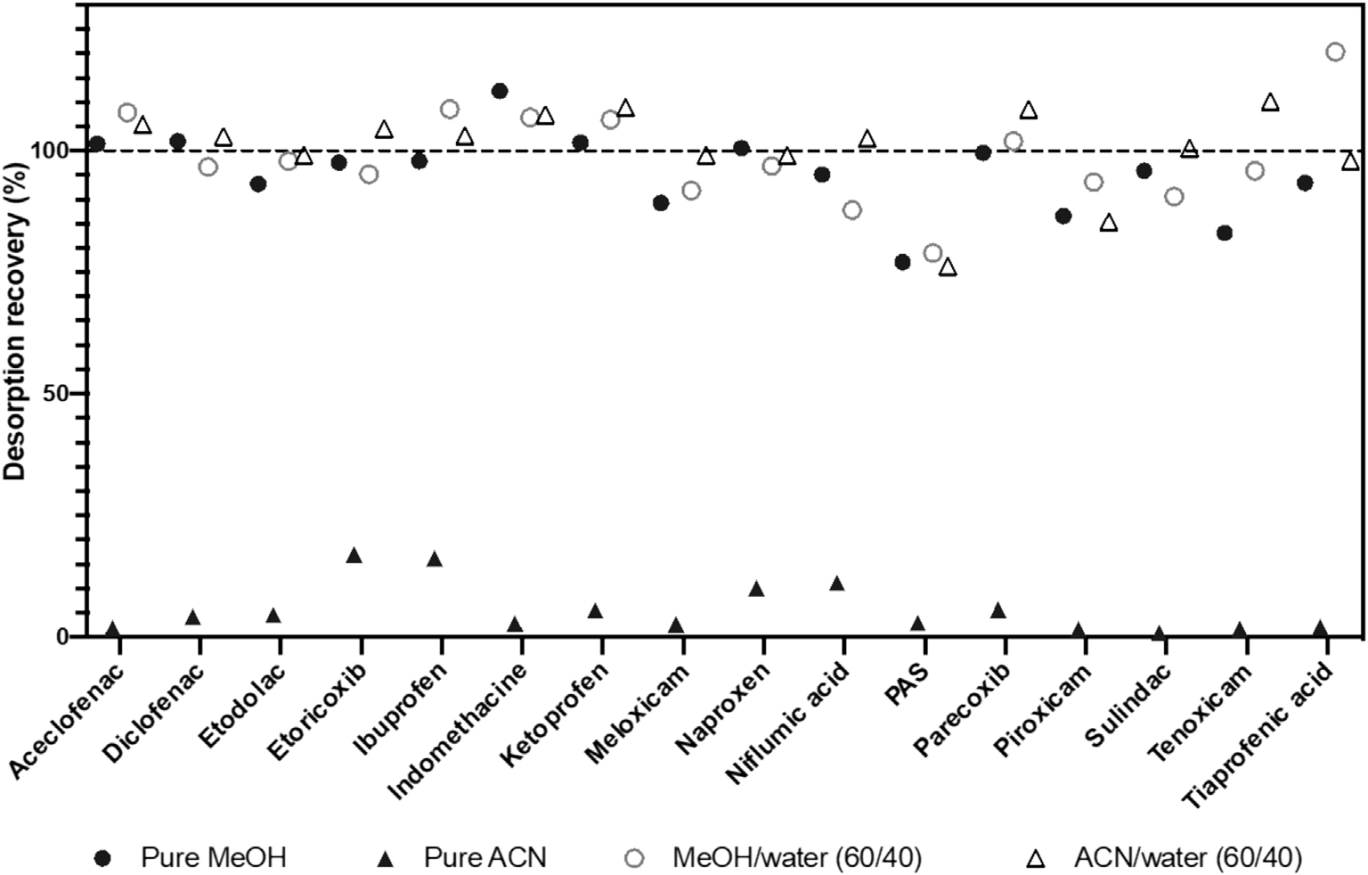
Desorption recoveries obtained with pure methanol (MeOH), pure acetonitrile (ACN), methanol and water mixture (MeOH/water), and acetonitrile and water mixture (ACN/water). PAS, para‐aminosalicylic acid.

Four different mixtures were compared for the desorption of NSAIDs from the DBS samples (Figure 1). The mean desorption recoveries for the 16 compounds obtained with pure MeOH, MeOH/water, ACN/water and pure ACN were (mean+/− sd) 95.4% ± 8.4%, 98.6% ± 9.9%, 100.7% ± 8.8% and 5.7% ± 5.4%, respectively. Due to very low values of the desorption recoveries, the ACN was excluded at this time. For all other mixtures, it was observed that after the resuspension of the dry residue the solution was not clear and slightly coloured in red. According to a previous work the mixture ACN/water was selected, and a LLE step using chloroform was added to obtain a cleaner sample.^6^ This additional LLE step increased the signal of NSAIDs by 30%. This value was determined by comparing area ratio of IS with and without LLE. The mean LLE recovery of the 16 NSAIDs was 84.5% ± 11.1% (Table S2).

Concerning the validation of the method, the carry‐over was considered insignificant because the highest value was 1.6% for piroxicam. The selectivity was validated for the 16 NSAIDs and ibuprofen metabolites. The calibration curves were constructed by plotting the ratio of the peak area of NSAIDs to that of the IS against the concentration of the calibration standards. A weighted linear least square regression was applied (Table 1). The LLOQ was defined as the lowest calibration standard. Limits of detection (LOD) ranged from 4.10^−5^ μg/mL (niflumic acid) to 1.10^−2^ μg/mL (ibuprofen) (Table 1). For all NSAIDs, accuracy and imprecision were within 15% for the 3 levels of QC and lower than 20% for the LLOQ (see Table S3). The matrix effect ranged from −33% to +39% depending on NSAIDs (see Table S2).

**TABLE 1:** Limit of detection (LOD), range of calibration curves and weighting factor for NSAIDs quantification in dried blood spot (DBS).

NSAIDs	LOD (μg/mL)	Calibration range (μg/mL)	Internal standard	Weighting
Aceclofenac	1.10^−3^	0.05–20	Diclofenac D_4_	1/x
Diclofenac	1.10^−3^	0.05–20	Diclofenac D_4_	1/x^2^
Etodolac	5.10^−4^	0.05–20	Sulindac D_6_	1/x^2^
Etoricoxib	2.10^−4^	0.02–5	Ketoprofen D_3_	1/x^2^
Hydroxyibuprofen	3.10^−3^	0.02–10	Ibuprofen ^13^C_6_	1/x
Ibuprofen	1.10^−2^	0.1–50	Ibuprofen ^13^C_6_	1/x
Ibuprofen glucuronide	3.10^−3^	0.02–10	Ibuprofen ^13^C_6_	1/x
Indomethacin	4.10^−3^	0.05–20	Diclofenac D_4_	1/x^2^
Ketoprofen	5.10^−4^	0.05–20	Ketoprofen D_3_	1/x^2^
Meloxicam	1.10^−4^	0.02–5	Sulindac D_6_	1/x
Naproxen	6.10^−3^	0.1–50	Ketoprofen D_3_	1/x
Niflumic acid	4.10^−5^	0.02–5	Diclofenac D_4_	1/x^2^
PAS	4.10^−4^	0.1–50	Sulfasalazin D_4_	1/x
Parecoxib	9.10^−4^	0.02–5	Ketoprofen D_3_	1/x
Piroxicam	2.10^−4^	0.02–5	Piroxicam D_3_	1/x^2^
Sulindac	9.10^−4^	0.05–20	Sulindac D_6_	1/x
Tenoxicam	3.10^−4^	0.02–5	Sulfasalazin D_4_	1/x
Tiaprofenic acid	7.10^−4^	0.1–50	Diclofenac D_4_	1/x

*Note*: For all NSAIDs, the quadratic model was used for the regression.

Abbreviation: PAS, para‐aminosalicylic acid.

### 3.2 Application of UTMB® samples

Five different NSAIDs were identified in the 56 positive DBS samples among 112 samples collected (39 women and 73 men): ibuprofen or metabolite (*n* = 45), diclofenac (*n* = 10, concentrations measured ranged from <LLOQ to 0.4 μg/mL), ketoprofen (*n* = 3, concentrations measured ranged from <LLOQ to 0.14 μg/mL), naproxen (*n* = 3, concentrations measured ranged from <LLOQ to 40.9 μg/mL) and niflumic acid (*n* = 2, concentrations measured <LLOQ). In six cases, more than one NSAIDs was detected in the same DBS sample (ibuprofen and diclofenac, naproxen or ketoprofen). Table 2 reports the data concerning ibuprofen and its metabolites for positive samples. Some of these results have previously been published by our lab.^4^ In two cases (trailers 44 and 45) ibuprofen was not detected despite the presence of hydroxyibuprofen and in two other cases (trailers 42 and 43) it was the opposite (Table 2). However, in the majority of cases, hydroxyibuprofen was detectable but not quantifiable, whereas ibuprofen could be quantified. Ibuprofen glucuronide and hydroxyibuprofen glucuronide were detected only when the ibuprofen concentration was greater than 3.3 μg/mL. Carboxyibuprofen was detected only when the ibuprofen concentration was higher than 0.45 μg/mL.

**TABLE 2:** Samples from Ultra‐Trail du Mont‐Blanc® (UTMB®) 2021 and 2022 trail runners in which ibuprofen and/or at least one of its metabolites has been identified.

Trailer	Ibu	HO‐Ibu	COOH‐Ibu	Ibu‐Glu	HO‐Ibu‐Glu	Trailer	Ibu	HO‐Ibu	COOH‐Ibu	Ibu‐Glu	HO‐Ibu‐Glu
1	31.50	5.0	d	1.50	d	24	0.16	>LOD	‐	‐	‐
2	21.10	1.9	d	0.66	d	25	0.15	>LOD	‐	‐	‐
3	20.10	2.9	d	1.05	d	26	0.14	>LOD	‐	‐	‐
4	19.10	6.6	d	1.12	d	27	0.14	>LOD	‐	‐	‐
5	15.60	6.2	d	0.88	d	28	0.11	>LOD	‐	‐	‐
6	8.70	0.6	d	0.27	d	29	0.11	>LOD	‐	‐	‐
7	3.30	1.3	d	0.09	d	30	0.10	>LOD	‐	‐	‐
8	1.32	0.03	d	‐	‐	31	>LOD	>LOD	‐	‐	‐
9	1.31	0.04	d	‐	‐	32	>LOD	>LOD	‐	‐	‐
10	0.67	0.04	d	‐	‐	33	>LOD	>LOD	‐	‐	‐
11	0.52	0.03	d	‐	‐	34	>LOD	>LOD	‐	‐	‐
12	0.45	0.03	d	‐	‐	35	>LOD	>LOD	‐	‐	‐
13	0.29	>LOD	‐	‐	‐	36	>LOD	>LOD	‐	‐	‐
14	0.29	>LOD	‐	‐	‐	37	>LOD	>LOD	‐	‐	‐
15	0.25	>LOD	‐	‐	‐	38	>LOD	>LOD	‐	‐	‐
16	0.24	>LOD	‐	‐	‐	39	>LOD	>LOD	‐	‐	‐
17	0.24	>LOD	‐	‐	‐	40	>LOD	>LOD	‐	‐	‐
18	0.23	0.04	‐	‐	‐	41	>LOD	>LOD	‐	‐	‐
19	0.22	>LOD	‐	‐	‐	42	>LOD	‐	‐	‐	‐
20	0.22	>LOD	‐	‐	‐	43	>LOD	‐	‐	‐	‐
21	0.20	0.02	‐	‐	‐	44	‐	>LOD	‐	‐	‐
22	0.19	>LOD	‐	‐	‐	45	‐	>LOD	‐	‐	‐
23	0.18	>LOD	‐	‐	‐						

*Note*: Concentrations are expressed in μg/mL. Lower limit of quantification (LLOQ) for ibuprofen was 0.1 μg/mL, for hydroxyibuprofen 0.02 μg/mL and for ibuprofen glucuronide 0.1 μg/mL. LOD for ibuprofen was 0.01 μg/mL, for hydroxyibuprofen 0.003 μg/mL and for ibuprofen glucuronide 0.02 μg/mL. LLOQ and LOD were not determined for COOH‐Ibu and HO‐Ibu‐Glu without pure compound available. *: Previously published results from our laboratory (see reference 4).

Abbreviations: ‘‐’, not detected; COOH‐Ibu, carboxyibuprofen; d, detected; HO‐ibu, hydroxyibuprofen; HO‐Ibu‐Glu, hydroxyibuprofen glucuronide; Ibu, ibuprofen; Ibu‐Glu, ibuprofen glucuronide.

### 3.3 Experimental kinetic study

**FIGURE 2:**
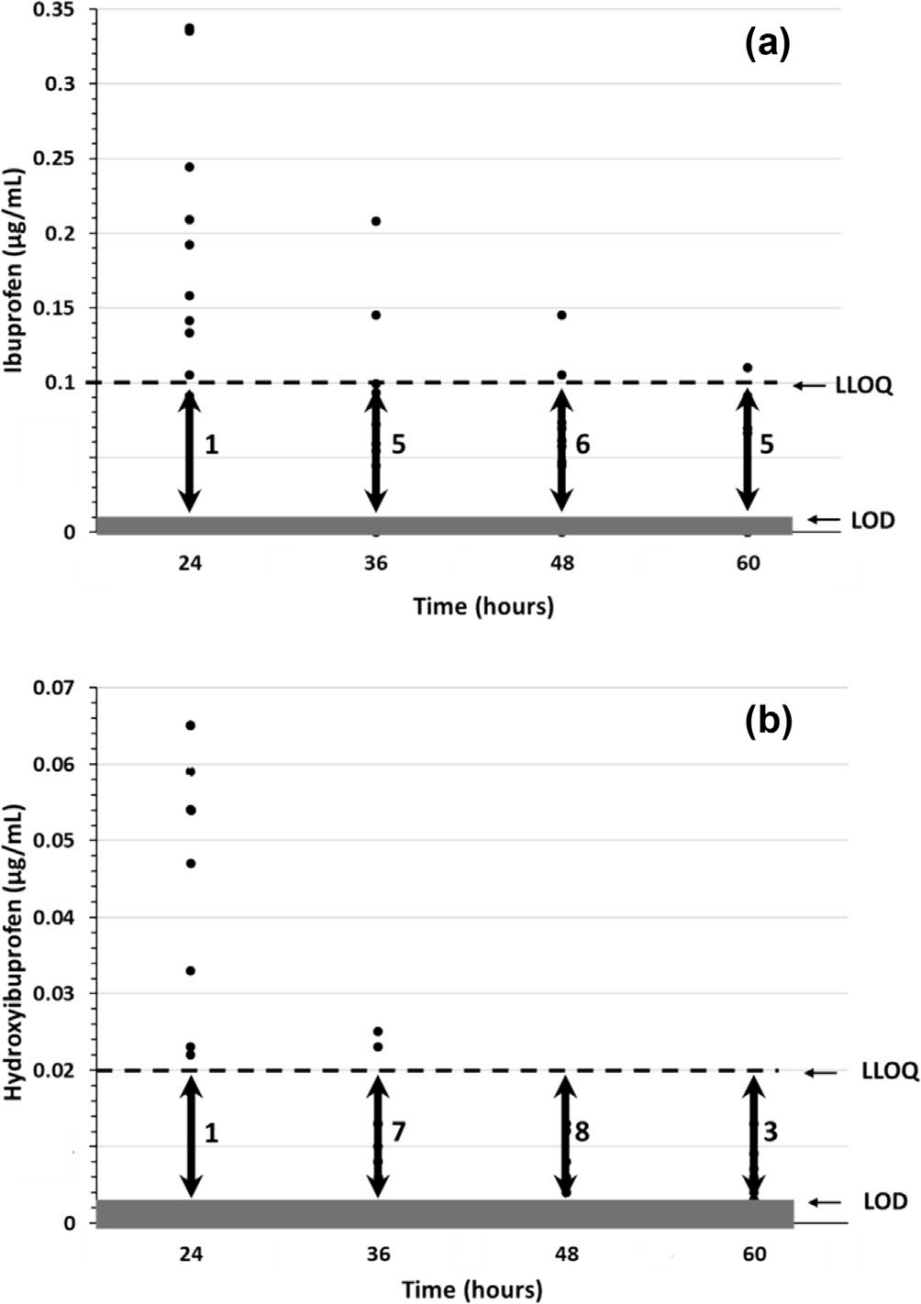
Late elimination phase of ibuprofen (a) and hydroxyibuprofen (b) from dried blood spot (DBS) in 10 healthy volunteers after an intake of 400 mg per os of ibuprofen. The number indicates the points between lower limit of quantification (LLOQ) (ibuprofen: 0.1 μg/mL, hydroxyibuprofen: 0.02 μg/mL) and LOD (ibuprofen: 0.01 μg/mL, hydroxyibuprofen: 0.003 μg/mL).

Concentrations of ibuprofen and hydroxyibuprofen from the 10 control subjects during the late elimination phase are presented in Figure 2. Concentration of ibuprofen could be determined in nine, three, two and one out of the 10 samples at 24, 36, 48 and 60 h, respectively. Ibuprofen was detectable but not quantifiable in one, five, six and five out of the 10 samples at 24, 36, 48 and 60 h, respectively. The results for hydroxyibuprofen showed that the number of quantifiable (LLOQ = 2.10^−2^ μg/mL) and detectable (LOD = 3.10^−3^ μg/mL) samples was equal or lower than ibuprofen whatever the sampling time. Carboxyibuprofen was detected in 10, three, two and two out of the 10 samples at 24, 36, 48 and 60 h, respectively. Ibuprofenglucuronide and hydroxyibuprofen glucuronide were never detected in these samples.

### 3.4 Pharmacokinetic modelling and simulations

**FIGURE 3:**
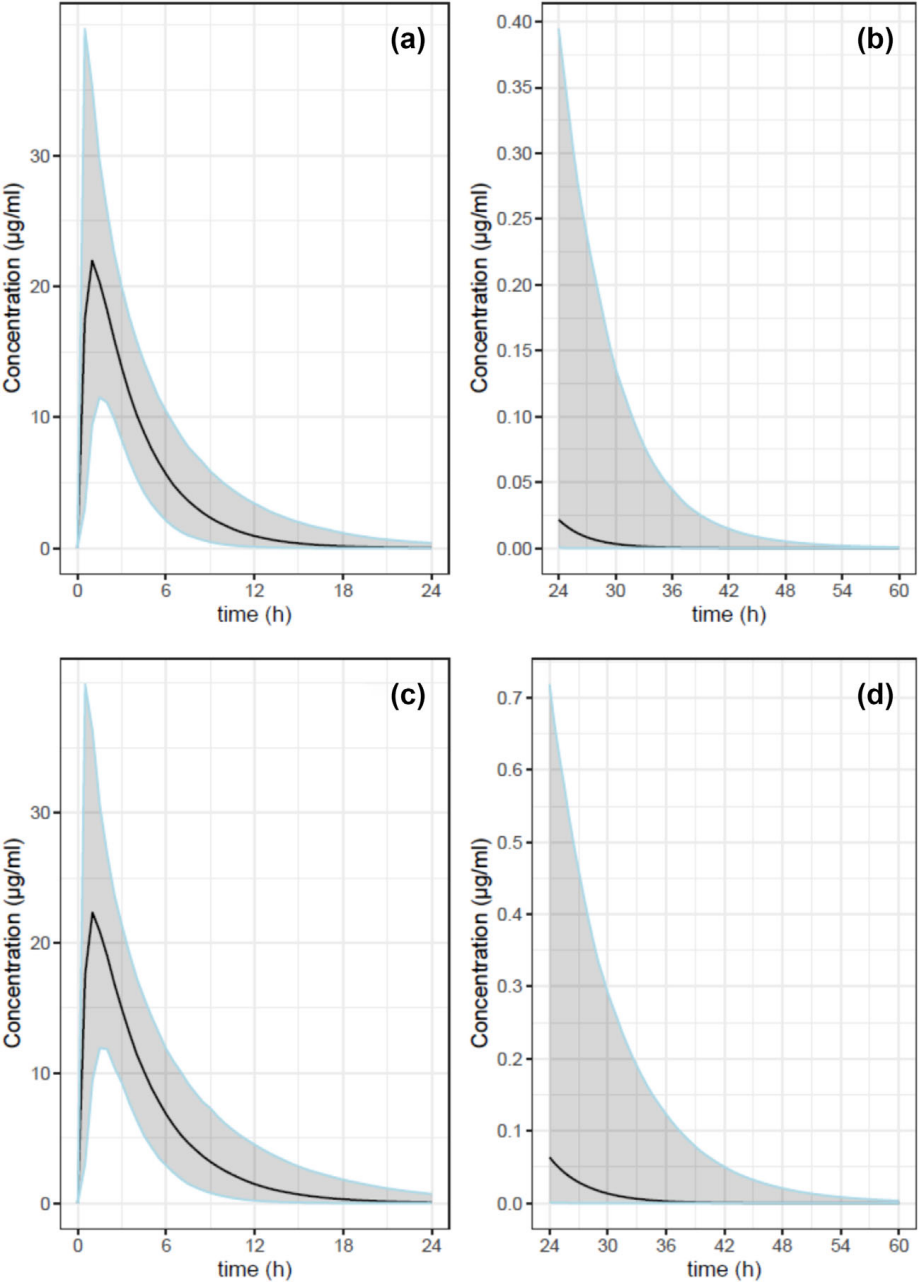
Concentration of ibuprofen in virtual fasted subjects after an intake per os of 400 mg using pharmacokinetic modellings. (a) (0–24 h) and (b) (24–60 h): hypothesis with a standard clearance. (c) (0–24 h) and (d) (24–60 h): hypothesis with a clearance decreased by 20%. The grey zone indicates the concentrations between the quartile 5 and the quartile 95. The black line corresponds to the quartile 50.

The blood concentrations of the 1,000 virtual patients generated (sex ratio of 1) from the pharmacokinetic model are presented in Figure 3. For example, quartile 5 (Q5) and quartile 95 (Q95) of the simulated patients showed ibuprofen concentrations of 0.13 and 395 ng/mL at 24 h and of <0.001 and 5.1 ng/mL at 48 h without modification of the total clearance. With a 20% reduction of total clearance (scenario 2), the simulated concentrations were 0.87 and 718 ng/mL at 24 h and <0.001 and 20 ng/mL at 48 h, for Q5 and Q95, respectively.

**FIGURE 4:**
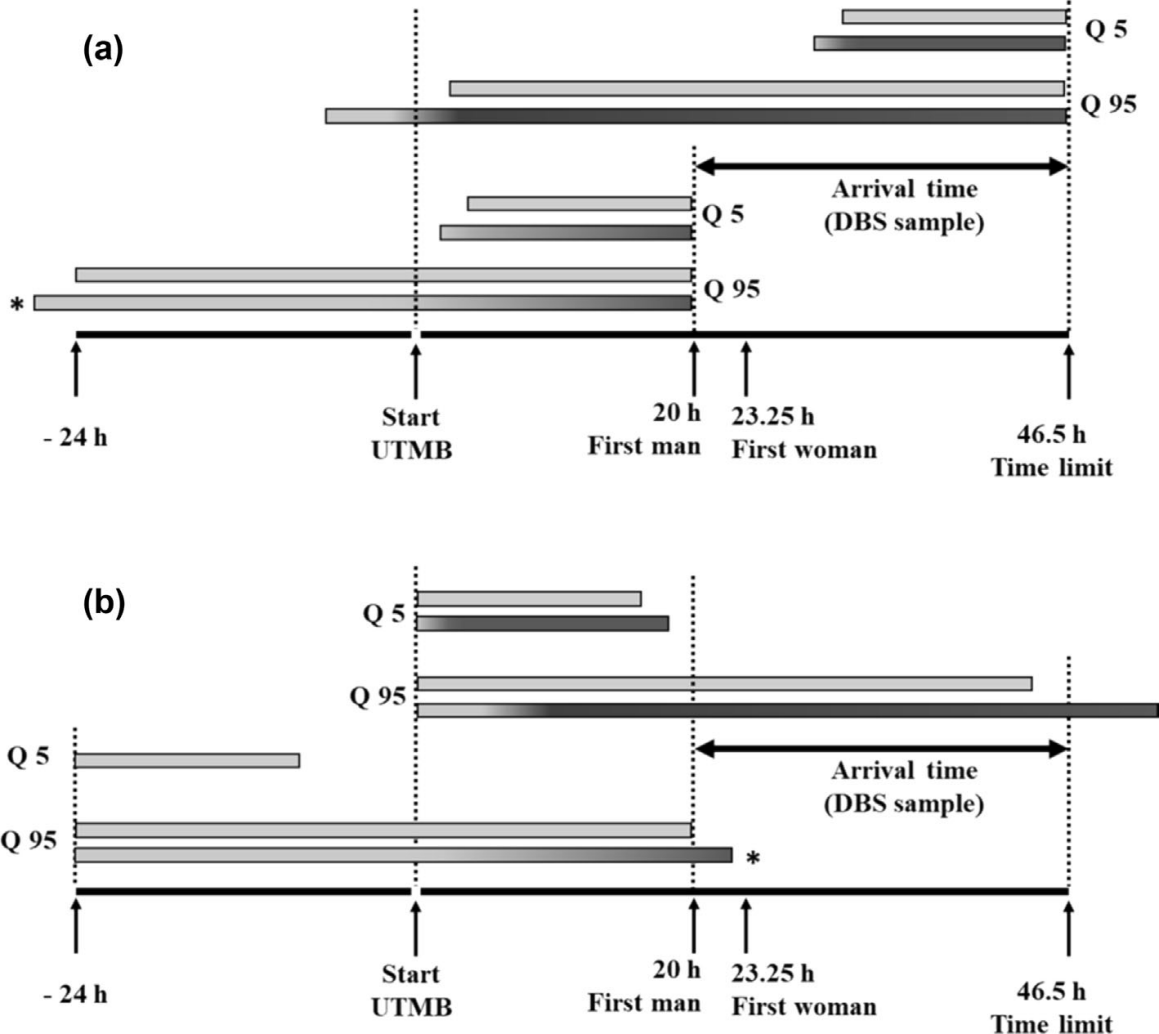
Detection windows of ibuprofen proposed from pharmacokinetic model after an oral intake of 400 mg and considering the limit of detection of ibuprofen from dried blood spot (DBS) sample at 0.01 μg/mL. Blood sample was collected when the trailer finished the race. (a) Detection windows calculated from the end of the trail, indicating earliest drug intake that can be detected. (b) Detection windows if the trailer takes ibuprofen at the time of departure or a day before. Grey: standard total clearance (scenario 1). From grey to dark grey: progressive reduction in the total clearance up to 20% (scenario 2). *: in this case ibuprofen is taken 1 day before, with the modification of the total clearance only the after departure from Ultra‐Trail du Mont‐Blanc® (UTMB®) (scenario 2bis). Scale: 24 h = 6 cm. Quartile 5 (Q5). Quartile 95 (Q95).

Considering the LOD of ibuprofen (0.01 μg/mL) and based on the model with the standard clearance, the ibuprofen could be detected during 16 to 44 h after the intake for the quantile 5 (Q5) and 95 (Q95), respectively. With the scenario 2 (total clearance decreasing of 20%), the detection may occur during 18 to 53 h after the intake of ibuprofen for Q5 and Q95, respectively. Based on the PK model, with the standard clearance and for the Q5, only recent intake (during 16 h) could be identified with certainty. Inversely, based on Q95, the detection window was assessed to 44 h corresponding to the day before and the race for the first trailers and almost corresponding to the race for the last finisher (Figure 4). In 2022, the best trail runners covered the distance of UTMB® in 20 h and the maximum time allowed for the entire UTMB® race was set at 46.5 h.

## 4 DISCUSSION

The developed method has been validated for the quantification of 16 NSAIDs and two ibuprofen metabolites in DBS. LODs vary greatly from one compound to another with ibuprofen having the highest value at 0.01 μg/mL by using 10 μL of blood spot. The majority of DBS‐based methods reported for therapeutic‐drug monitoring used pure MeOH as desorption solvent and do not perform an additional extraction phase.^11^ However, in the present case, the addition of a LLE step increases the sensitivity by reducing the matrix effect, with the main drawback of inducing a longer sample preparation. To our knowledge, only one article presents the quantification of NSAIDs in DBS.^12^ It was not a method dedicated to NSAIDs because only three NSAIDs (etoricoxib, indomethacin and sulindac) studied in the present work were included in a list of 425 compounds.

Although several NSAIDs were detected from trailer samples, ibuprofen was the drug most frequently identified. Thus, we focused our additional work on this compound. The concentrations of ibuprofen from trailers ranged from 0.1 to 31.5 μg/mL, with in most cases concentrations below 1 μg/mL. In 15 cases, ibuprofen or hydroxyibuprofen were detectable but not quantifiable (Table 2). We further wanted to know how to interpret these data in relation with the Quartz rules which stipulate that it is forbidden to consume within 24 h before the start and during the trail NSAIDs regardless of the mode of administration. According to the 2022 results, the arrival window is between 20 and 46.5 h after the start of the race. Thus, it was necessary to be able to determine if the trailer consumed ibuprofen in the 44 to 70.5 h before DBS collection performed at the end of the trail. In a previous work, we have shown that it is not recommended to rely on the declaration of consumption from the trailers because of an underestimation.^4^

Results obtained from 10 control subjects after an oral intake of 400 mg of ibuprofen shown the presence (quantifiable or detectable) of the drug in all cases after 24 h, and in 8, 8 and 6 cases after 36, 48 and 60 h, respectively. Thus, this first approach based on an experimental kinetic study indicated that the absence of ibuprofen in DBS samples at the finish of the race allowed to exclude an intake during the race but do not exclude an intake 24 h before the start, at least for the fastest trailers. In addition, considering the maximum delay (time barrier), only the second part of the race (the last 24 h) may be monitored with certainty. The monitoring of ibuprofen metabolites does not bring additional information. Hydroxyibuprofen was not detected in all samples at 60 h, carboxyibuprofen was systematically detected only at 24 h and ibuprofen glucuronide and hydroxyibuprofen glucuronide were never detected from 24 to 60 h. The use of a pharmacokinetic model allowed to test a large number of subjects and take into account interindividual variability. Moreover, the model made it possible to compare two scenarios: trailers with standard total clearance and a gradual decrease in the total clearance up to 20% from the beginning of the race. This hypothesis was considered because it was demonstrated that long and intensive physical activities alter the pharmacokinetics of drugs.^13^, ^14^, ^15^, ^16^ The effects are complex and depend on physiological changes (redistribution of blood flow, increase in cardiac output, loss of water from plasma into tissues, etc.) and on kinetic properties of the drugs (hepatic extraction, renal elimination, plasma protein binding, etc.). In the present work, a reduction in the total clearance of ibuprofen was hypothesized as the main consequence of the redistribution of the blood flow towards the muscles to the detriment of the liver and kidneys. For ibuprofen, only a decrease in free metabolic clearance has been shown in relation to the reduction in the hepatic blood flow, and this drug is eliminated unchanged by renal filtration only for 10% in the urine.^17^, ^18^ For these both reasons, an impact limited to 20% on the total clearance of ibuprofen was considered as a realistic hypothesis. The estimation of the detection windows based on the pharmacokinetic model and the LOD of ibuprofen made it possible to show that its detection is only possible over a period of time less than the maximum 70.5 h (24 h before departure and the limit time of 46.5 h). This approach, however, allows us to estimate that the detection of an ibuprofen intake during the race remains probable at least for the first trail runners (in 2022, the 10th man arrived in 22 h and the 10th woman in 28 h) on the basis of Q50 (scenario 1: 26 h and scenario 2: 31 h). Consumption of ibuprofen is probably most frequently performed during the race because a significant and relatively rapid pain intensity reduction was observed throughout the 6–8 h period after intake.^2^, ^19^ Indeed, due to a short half‐life, ibuprofen must be taken frequently so that blood concentrations are within the broad therapeutic zone of 10 to 50 μg/mL.^18^ Taking ibuprofen within 24 h before the race will not allow this concentration zone to be reached during the race and will therefore have no impact on performance.

It is interesting to note that results obtained from 10 control subjects are in accordance with the scenario 1 of the model, as the measured concentrations were between the median concentrations (Q50) and the Q95 of the model for the different times studied. The fact that ibuprofen was below LLOQ and even undetectable in some samples for the later times of the kinetic is also consistent with the pharmacokinetic model. The capacity of the method to detect ibuprofen 70.5 h (day before + race time to 46.5 h) after the intake would be 0% according to the pharmacokinetics model and the LOD of the assay. The experimental data obtained in control subjects do not allow us to exclude, by extrapolating the results at 60 h, that for a very small number of individuals, ibuprofen would still be detectable. Conversely, although unlikely, it cannot be excluded that for the first trail runners the presence of ibuprofen was the consequence of an intake of the drug few hours before the 24 h preceding the start of the race. The absence of ibuprofen does not allow us to exclude an intake more than 16 h before the collection of DBS according to the scenario 1 of the pharmacokinetic model (standard clearance, Q5) and 36 h according to experimental data from control subjects.

To increase the detection window, it would be necessary to have a more sensitive analytical method than that proposed here and/or to increase the volume of blood sample (assuming a better signal/noise ratio can be obtained). DBS sampling has obvious practical advantages but only allows a very small volume of blood to be available. A blood sample of several millilitres is extremely difficult to implement for practical reasons in the context of trails (need qualified staff, dedicated room, rapid delivery of blood samples while respecting temperature conditions, etc.). A urine sample could constitute a relevant alternative matrix in terms of detection time. But this matrix has also some disadvantages such as infrastructure for the collection, dehydration of runners with delayed urine output, possible adulteration or falsification of the sample and sample storage temperature. Oral fluid could also be an alternative, but in a previous study, we showed that the detection windows were much shorter than with DBS.^4^ So, taking all these points into account, DBS seems to remain the most interesting type of sampling. To compensate for a detection window, which can be relatively short, collecting several samples from the same trailer (at the departure and the arrival, e.g.) could be another alternative.

In this work, the real intake (experimental data) or hypothetical (model pharmacokinetic) was 400 mg of ibuprofen in a single dose. The conclusions would be different in the case of a higher dose or if repeated doses were taken. Likewise, the conclusions would be different with other NSAIDs, which would probably have larger detection windows. This could be explained by the fact, although having a short half‐life (such as diclofenac, ketoprofen, aceclofenac, niflumic acid), the LODs of the analytical method for these NSAIDs were lower, or because the elimination half‐lives of drugs are longer (such as meloxicam, naproxen, etoricoxib). Thus, the ibuprofen model can be considered as the ‘worst case’ with a compound with a short half‐life and the highest LOD.

## 5 CONCLUSION

A validated method for the quantification of several NSAIDs and two ibuprofen metabolites was proposed from DBS samples. An additional LLE step was added to improve LOD. We have focused on ibuprofen, the most NSAID detected in the samples of UTMB® 2021 and 2022 runners. An original approach coupling a pharmacokinetic model of ibuprofen adjusted to a population of ultra‐trailers and the LOD from DBS provided data to propose detection windows for this NSAID. It was demonstrated that the analysis of ibuprofen from a DBS does not make it possible to ensure compliance with the Quartz regulation with certainty, that is, the detection of ibuprofen during, at most, 70.5 h before the end of the race. It also showed the challenge of suggesting a time of intake if a very low concentration is detected. Thus, the detection of ibuprofen in the blood attests the very high probability to the non‐compliance with the rule. However, the non‐detection of ibuprofen did not exclude an intake during the prohibited period. Another approach may be to multiply the DBS samples by collecting a sample at the start and at the end of the race. Ultimately, the determination of a threshold concentration of positivity beyond which trail runners would be disqualified, as practiced in anti‐doping approach, would be unequivocal. However, it remains that indicating to runners that samples may be carried out constitutes a way of encouraging them to respect the rules.

## CONFLICT OF INTEREST STATEMENT

No potential conflict of interest was reported by the authors.

## Supporting information


**Table S1.** Details on the LC–MS/HRMS method used for the quantitative analysis of NSAIDs.
**Table S2.** Liquid–liquid extraction (LLE) recovery and matrix effect for each NSAID and ibuprofen metabolites.
**Table S3.** Accuracy and precision.
**Figure S1.** The decrease in clearance as a function of time is modelled by a sigmoid relationship according to the following equation: Cl = Clini * (1 ‐ Imax * tˆgamma / [tˆgamma + T50ˆgamma]). The Clini parameter described the initial clearance, Imax the maximal reduction of clearance (20%), T50 the time at which the clearance is reduced by half at 2 h (A ‐ scenario 2) and at 26 h (B ‐ scenario 2bis) of the maximal reduction, and gamma characterizes the sigmoidal shape (arbitrary equal to 1).
