# Supplementary material for: Quantitative analysis of nonsteroidal anti‐inflammatory drugs in dried blood spot from mountain ultra‐trail runners. Contribution of pharmacokinetic models for the interpretation of the results
Source: Drug Test Anal. 2024 Aug 15;17(6):825–33. doi: 10.1002/dta.3781 (PMC12151714; doi:10.1002/dta.3781)
Supplement: Supplementary file 1 — Table S1. Details on the LC–MS/HRMS method used for the quantitative analysis of NSAIDs. Table S2. Liquid–liquid extraction (LLE) recovery and matrix effect for each NSAID and ibuprofen metabolites. Table S3. Accuracy and precision. Figure S1. The decrease in clearance as a function of time is modelled by a sigmoid relationship according to the following equation: Cl = Clini * (1 ‐ Imax * tˆgamma / [tˆgamma + T50ˆgamma]). The Clini parameter described the initial clearance, Imax the maximal reduction of clearance (20%), T50 the time at which the clearance is reduced by half at 2 h (A ‐ scenario 2) and at 26 h (B ‐ scenario 2bis) of the maximal reduction, and gamma characterizes the sigmoidal shape (arbitrary equal to 1). [file DTA-17-825-s001.docx]

Table S1. Details on the LC-MS/HRMS method used for the quantitative analysis of NSAIDs

| **Liquid chromatography** | | | |
| --- | --- | --- | --- |
| **Column** | Accucore AQ (150 x 2.1 mm; 2.6 μm) (Thermo Scientific, USA) | | |
| **Mobile phase** | A: 0.1% acetic acid in acetonitrile  B: 0.1% acetic acid in water | | |
| **Gradient** | 0 min: 25% A  1 min: 25% A  2.9 min: 95% A  6.4 min: 95% A  6.5 min: 25% A  10.4 min: 25% A | | |
| **Flow rate** | 200 µL/min | | |
| **Mass spectrometry** | | | |
| **NSAID** | **(i.m) m/z** | **NSAID** | **(i.m) m/z** |
| Aceclofenac | (+) 354.02944 | Niflumic Acid | (+) 283.06889 |
| Diclofenac | (+) 296.02396 | PAS | (+) 154.04987 |
| Etodolac | (+) 288.15942 | Parecoxib | (+) 371.10600 |
| Etoricoxib | (+) 359.06155 | Piroxicam | (+) 332.06995 |
| Ibuprofen | (-) 205.12340 | Sulindac | (+) 357.09552 |
| Indomethacine | (+) 358.08406 | Tenoxicam | (+) 338.02637 |
| Ketoprofen | (+) 255.10157 | Tiaprofenic Acid | (+) 261.05799 |
| Meloxicam | (+) 352.04202 | Hydroxyibuprofen | (-) 221.11832 |
| Naproxen | (-) 229.08702 | Ibuprofen glucuronide | (-) 381.15549 |
| Diclofenac D_4_ | (+) 300.04907 | Piroxicam D_3_ | (+) 335.08878 |
| Ibuprofen ^13^C_6_ | (-) 211.14353 | Sulfasalazine D_4_ | (+) 403.10087 |
| Ketoprofen D_3_ | (+) 258.12040 | Sulindac D_6_ | (+) 363.13318 |

i.m: ionization mode; PAS: Para-aminosalicylic acid

Table S2. Liquid-liquid extraction (LLE) recovery and matrix effect for each NSAID and ibuprofen metabolites.

| **NSAID** | **LLE recovery (%)** | **Matrix effect at QC low (%)** | **Matrix effect at QC high (%)** |
| --- | --- | --- | --- |
| **Aceclofenac** | 93.7 | -16.9 | -3.1 |
| **Diclofenac** | 93.4 | -27.9 | -6.1 |
| **Etodolac** | 95.6 | 14.0 | 3.4 |
| **Etoricoxib** | 81.7 | -5.2 | 0.6 |
| **Hydroxyibuprofen** | 82.5 | -4.8 | -12.9 |
| **Ibuprofen** | 91.5 | -1.3 | -32.6 |
| **Ibuprofen glucuronide** | 81.2 | -1.8 | -9.8 |
| **Indomethacine** | 86.1 | -13.5 | -0.3 |
| **Ketoprofen** | 76.9 | -15.9 | -2.8 |
| **Meloxicam** | 93.2 | 35.3 | 4.3 |
| **Naproxen** | 87.8 | -23.8 | -1.8 |
| **Niflumic Acid** | 78.1 | -18.1 | -3.8 |
| **PAS** | 88.2 | -26.2 | -5.7 |
| **Parecoxib** | 87.5 | -16.2 | -5.8 |
| **Piroxicam** | 50.1 | 39.0 | -6.0 |
| **Sulindac** | 84.5 | -8.1 | -5.5 |
| **Tenoxicam** | 80.4 | 15.5 | 8.8 |
| **Tiaprofenic Acid** | 79.5 | -10.9 | -1.9 |

PAS: Para-aminosalicylic acid

Table S3. Accuracy and precision

QC: quality control; LLOQ: low limit of quantification; CV: coefficient of variation

| **NSAID** | **Theoretical values (µg/mL)** | | | | **Within-day accuracy (%)** | | | | **Between-day accuracy (%)** | | | | **Within-day precision (CV %)** | | | | **Between-day precision (CV %)** | | | | |  |
| --- | --- | --- | --- | --- | --- | --- | --- | --- | --- | --- | --- | --- | --- | --- | --- | --- | --- | --- | --- | --- | --- | --- |
|  | **LLOQ** | **QC low** | **QC medium** | **QC high** | **LLOQ** | **QC low** | **QC medium** | **QC high** | **LLOQ** | **QC low** | **QC medium** | **QC high** | **LLOQ** | **QC low** | **QC medium** | **QC high** | | **LLOQ** | **QC low** | **QC medium** | **QC high** | |
| **Aceclofenac** | 0.05 | 0.09 | 0.95 | 9.7 | 112.0 | 96.9 | 91.2 | 109.5 | 112.7 | 103.3 | 98.1 | 112.3 | 3.6 | 9.5 | 3.4 | 3.9 | | 8.00 | 2.84 | 3.39 | 4.80 | |
| **Diclofenac** | 0.05 | 0.09 | 0.95 | 9.7 | 110.8 | 96.4 | 100.5 | 107.5 | 116.7 | 96.3 | 98.8 | 107.4 | 4.7 | 6.4 | 2.8 | 2.3 | | 10.04 | 3.53 | 4.82 | 2.20 | |
| **Etodolac** | 0.05 | 0.09 | 0.95 | 9.7 | 95.6 | 99.6 | 98.3 | 100.2 | 94.0 | 92.2 | 99.6 | 102.4 | 11.0 | 7.2 | 5.5 | 6.4 | | 7.67 | 6.38 | 5.38 | 6.53 | |
| **Etoricoxib** | 0.02 | 0.12 | 0.41 | 3.7 | 89.0 | 92.3 | 103.9 | 100.6 | 118.3 | 86.9 | 99.2 | 97.4 | 12.8 | 5.8 | 4.3 | 3.4 | | 12.91 | 5.28 | 11.97 | 5.07 | |
| **Hydroxy**  **ibuprofen** | 0.02 | 0.30 | 2.80 | 8.80 | 94.0 | 99.1 | 98.8 | 105.3 | 106.7 | 99.2 | 94.9 | 97.7 | 4.5 | 6.8 | 4.0 | 6.0 | | 7.2 | 4.5 | 9.8 | 7.6 | |
| **Ibuprofen** | 0.10 | 0.28 | 2.90 | 33.0 | 98.2 | 94.1 | 89.3 | 110.8 | 89.7 | 90.8 | 104.4 | 109.5 | 2.6 | 7.6 | 0.6 | 1.6 | | 6.14 | 6.17 | 10.37 | 2.36 | |
| **Ibuprofen glucuronide** | 0.02 | 0.30 | 2.80 | 8.80 | 103.0 | 95.5 | 94.2 | 101.5 | 110.0 | 99.1 | 94.7 | 98.2 | 5.5 | 5.6 | 9.7 | 6.3 | | 7.9 | 7.9 | 13.8 | 2.8 | |
| **Indomethacine** | 0.05 | 0.09 | 0.95 | 9.7 | 109.2 | 86.2 | 106.4 | 99.0 | 110.7 | 91.5 | 104.6 | 103.2 | 3.8 | 4.8 | 3.7 | 4.7 | | 11.62 | 7.81 | 6.06 | 5.41 | |
| **Ketoprofen** | 0.05 | 0.09 | 0.95 | 9.7 | 109.6 | 106.2 | 104.7 | 114.4 | 107.3 | 103.3 | 103.6 | 111.3 | 3.5 | 2.4 | 1.0 | 0.6 | | 2.85 | 2.15 | 1.90 | 1.93 | |
| **Meloxicam** | 0.02 | 0.12 | 0.41 | 3.7 | 86.0 | 98.2 | 100.1 | 98.2 | 95.0 | 96.9 | 103.6 | 91.9 | 18.1 | 8.7 | 4.1 | 3.9 | | 5.26 | 4.73 | 2.83 | 3.63 | |
| **Naproxen** | 0.10 | 0.28 | 2.90 | 33.0 | 104.4 | 94.6 | 93.3 | 87.0 | 100.7 | 96.8 | 101.3 | 96.4 | 5.1 | 4.1 | 5.2 | 5.0 | | 11.77 | 4.49 | 6.73 | 2.35 | |
| **Niflumic Acid** | 0.02 | 0.12 | 0.41 | 3.7 | 103.0 | 106.5 | 109.8 | 112.4 | 113.3 | 99.2 | 98.8 | 92.3 | 8.8 | 11.8 | 4.1 | 4.6 | | 2.55 | 3.66 | 2.15 | 1.62 | |
| **PAS** | 0.10 | 0.28 | 2.90 | 33.0 | 108.0 | 98.4 | 111.4 | 88.0 | 94.0 | 106.5 | 109.0 | 101.7 | 11.9 | 4.0 | 8.1 | 8.8 | | 15.67 | 10.07 | 14.75 | 14.99 | |
| **Parecoxib** | 0.02 | 0.12 | 0.41 | 3.7 | 90.0 | 90.5 | 99.4 | 101.0 | 105.0 | 99.7 | 114.2 | 100.8 | 11.1 | 8.7 | 2.1 | 2.7 | | 8.25 | 3.48 | 9.10 | 3.97 | |
| **Piroxicam** | 0.02 | 0.12 | 0.41 | 3.7 | 88.0 | 95.8 | 96.3 | 100.5 | 116.7 | 95.3 | 104.7 | 99.4 | 11.1 | 6.9 | 1.0 | 2.4 | | 13.78 | 5.82 | 6.89 | 1.62 | |
| **Sulindac** | 0.05 | 0.09 | 0.95 | 9.7 | 106.8 | 101.1 | 93.0 | 97.7 | 113.0 | 98.1 | 96.2 | 103.3 | 8.6 | 2.8 | 2.4 | 4.9 | | 3.75 | 6.54 | 4.20 | 3.00 | |
| **Tenoxicam** | 0.02 | 0.12 | 0.41 | 3.7 | 94.0 | 95.0 | 89.8 | 100.3 | 106.7 | 104.2 | 104.3 | 97.0 | 11.5 | 3.7 | 2.2 | 7.5 | | 5.41 | 7.63 | 6.68 | 7.73 | |
| **Tiaprofenic Acid** | 0.10 | 0.28 | 2.90 | 33.0 | 111.4 | 105.4 | 89.8 | 88.2 | 94.3 | 89.8 | 92.5 | 90.3 | 2.3 | 4.6 | 1.4 | 4.0 | | 4.01 | 3.78 | 6.33 | 3.09 | |

PAS: Para-aminosalicylic acid

Fig. S1. The decrease in clearance as a function of time is modeled by a sigmoid relationship according to the following equation:

Cl = Clini * (1 - Imax * tˆgamma / (tˆgamma + T50ˆgamma))

The Clini parameter described the initial clearance, Imax the maximal reduction of clearance (20 %), T50 the time at which the clearance is reduced by half at 2 h (A - scenario 2) and at 26 h (B - scenario 2bis) of the maximal reduction, and gamma characterizes the sigmoidal shape (arbitrary equal to 1).


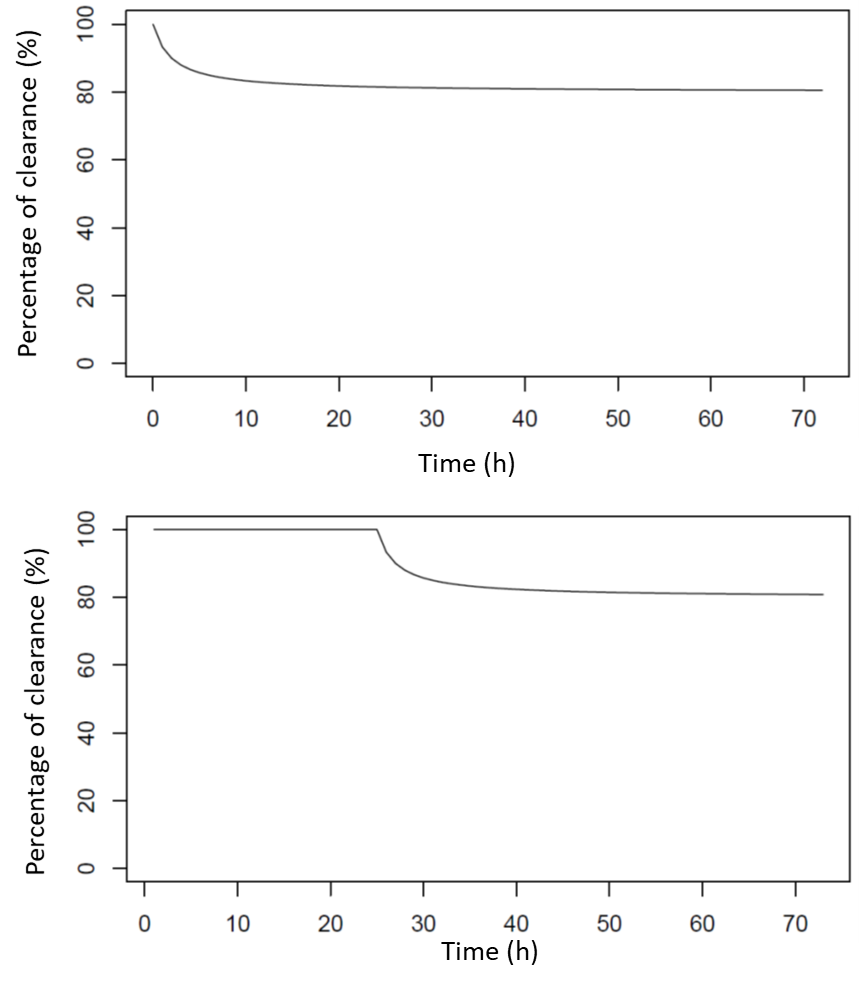


**A**

**B**
